# The effects of a firefighting simulation on the vascular and autonomic functions and cognitive performance: a randomized crossover study

**DOI:** 10.3389/fphys.2023.1215006

**Published:** 2023-09-21

**Authors:** Iara G. Teixeira, Marcio R. Verzola, Richard E. Filipini, Guilherme F. Speretta

**Affiliations:** ^1^ Post-Graduate Program in Neurosciences, Federal University of Santa Catarina, Florianópolis, Brazil; ^2^ Department of Physiological Sciences, Biological Sciences Centre, Federal University of Santa Catarina, Florianópolis, Brazil; ^3^ Military Firefighters Corps of Santa Catarina, Florianópolis, Brazil

**Keywords:** autonomic nervous system, carotid arteries, cognition, heart disease risk factors, heart rate, occupational health

## Abstract

**Introduction:** During firefighting, physical and cognitive demands increase. However, the stress inherent to these events can decrease cognitive performance and increase the risk of cardiovascular events in firefighters. Thus, this crossover study aimed to evaluate the effects of a firefighting Simulation on cognitive performance and vascular and autonomic functions in military firefighters.

**Methods:** Sixteen firefighters (37.8 ± 5.6 years) underwent anthropometry, mental health status, and sleep quality assessments. They randomly performed two interventions, Simulation (Firefighting tasks; 10.0 ± 1.1 min) and Control (rest for 10 min), on different days. After both interventions, cognitive performance was assessed using the Stroop Test, Paced Auditory Serial Addition Test, and Trail Making Test. Then, the vascular function was assessed using ultrasonography through the carotid artery reactivity to the cold pressor test. The arterial pressure, heart rate, and cardiac intervals were recorded before interventions. The cardiac intervals were also measured during the cold pressor test. Student’s t-test and Wilcoxon were used for comparisons between Control and Simulation and the analysis of variance for repeated measures was used for comparison over time during the cold pressor test. A significance level of *p* < 0.05 was adopted.

**Results:** Although the mean and maximum heart rate were higher before the Simulation (*p* < 0.0001), all the heart rate variability parameters (*p* > 0.05) and mean arterial pressure (*p* > 0.3795) were similar before the interventions. After Simulation, the cognitive performance was similar to Control (*p* > 0.05), except for the improvement in Stroop Test part B (*p* < 0.0001). After Simulation, carotid artery reactivity was attenuated (*p* < 0.0010). During the cold pressor test, the high-frequency band of the heart rate variability was lower after the Simulation (*p* < 0.0104).

**Discussion:** Although firefighting Simulation did not substantially change cognitive performance, the lower carotid artery reactivity and parasympathetic modulation to the heart during the cold pressor test may contribute to greater vulnerability to cardiovascular events in firefighters on duty.

## 1 Introduction

Stress situations trigger the increased activity of the sympathetic autonomic nervous system and the release of essential hormones such as adrenaline, noradrenaline, and cortisol (Ulrich-Lai and Herman, 2009). These changes trigger cardiovascular adjustments such as increased arterial pressure (AP) and platelet aggregation (Reid et al., 2009; Chen et al., 2016) and decreased baroreflex sensitivity and heart rate variability (HRV) (Adlan et al., 2018), preparing the individual for the defense. However, exposure to stress can increase the risk for cardiovascular events, especially in people with cardiovascular disorders (Steptoe and Kivimäki, 2012).

Acute physical and mental stress can also influence cognitive performance. Studies have shown worsening attention (Robinson et al., 2013; Sänger et al., 2014; Hemmatjo et al., 2018) and working memory (Zare et al., 2018; Hemmatjo et al., 2020) under stress conditions, while others have observed improvement in these outcomes (Greenlee et al., 2014). The improvement or deterioration in cognitive performance seems to depend on the magnitude of the stress and the complexity of the required cognitive functions (Arnsten, 2009). The cognitive performance depends on the interaction between the prefrontal cortex (Arnsten and Goldman-Rakic, 1998; Miller and Cohen, 2001) and responses modulated by the amygdala (Goldstein et al., 1996). While under basal conditions, the prefrontal cortex enables more complex cognitive responses, under stress, the responses may be more reflexive and faster but less specific (Arnsten, 2009; van Marle et al., 2009).

On the other hand, chronic stress seems to be a risk factor for cardiovascular disease and cognitive performance impairment. For instance, chronic stress is associated with a 40%–50% increase in the occurrence of coronary disease (Steptoe and Kivimäki, 2012). In addition, chronic stress seems to influence negatively other risk factors for cardiovascular disease and cognitive performance impairment, such as sleep quality (Taheri and Irandoust, 2020; Csipo et al., 2021; Hu et al., 2021) and mental health (Nicholson et al., 2006; Roest et al., 2010).

Exposure to stress can be frequent in some professions, such as firefighters. Situations such as seeing and caring for victims with lacerated bodies (Peterson et al., 2019), exposure to smoke (Hemmatjo et al., 2018), sleep disruption (Wolkow et al., 2016), risk of accidents at work (Yoon et al., 2016), excessive heat (Hemmatjo et al., 2017) and moral distress in decision-making (Lentz et al., 2021), can impact cardiovascular function and cognitive performance in firefighters. Indeed, cardiovascular diseases are the principal cause of death among firefighters, mainly associated with fire suppression (Kales et al., 2007). In addition, attenuating cognitive performance can affect work performance and increase the risk of accidents (Wadsworth et al., 2003). However, studies assessing cognitive performance in this population also showed conflicting results (Morley et al., 2012; Greenlee et al., 2014; Hemmatjo et al., 2020).

In this context, evaluating vascular and autonomic functions and cognitive performance in firefighters at rest and after a work activity with physical and mental demands becomes relevant. However, few studies have dedicated themselves to studying this theme. Considering the above, we aimed to assess the effects of firefighting Simulation on cognitive performance and vascular and autonomic functions in military firefighters.

## 2 Materials and methods

### 2.1 Participants

Eighteen males, who were part of the Military Firefighters Corps with at least 3 years of profession, were recruited. For convenience, the sample consisted of firefighters taking the sergeant training course. Exclusion criteria were: have visible or known cardiovascular, mental, or metabolic diseases; have infectious or inflammatory processes; have muscle or joint damage; smokers; or take a drug that could influence the analyzed variables. Two firefighters were excluded because they used drugs that could influence the variables assessed. One participant was excluded from the analysis of autonomic modulation during the cold pressor test (CPT) due to recording failure. We registered the protocol in the Brazilian Registry of Clinical Trials (ReBEC; n^o^. RBR-24db7p7). The Human Research Ethics Committee of the Federal University of Santa Catarina approved the experimental procedures (protocol n. 4.942.301). The participants signed the Free and Informed Consent Term.

### 2.2 Experimental protocol

**FIGURE 1:**
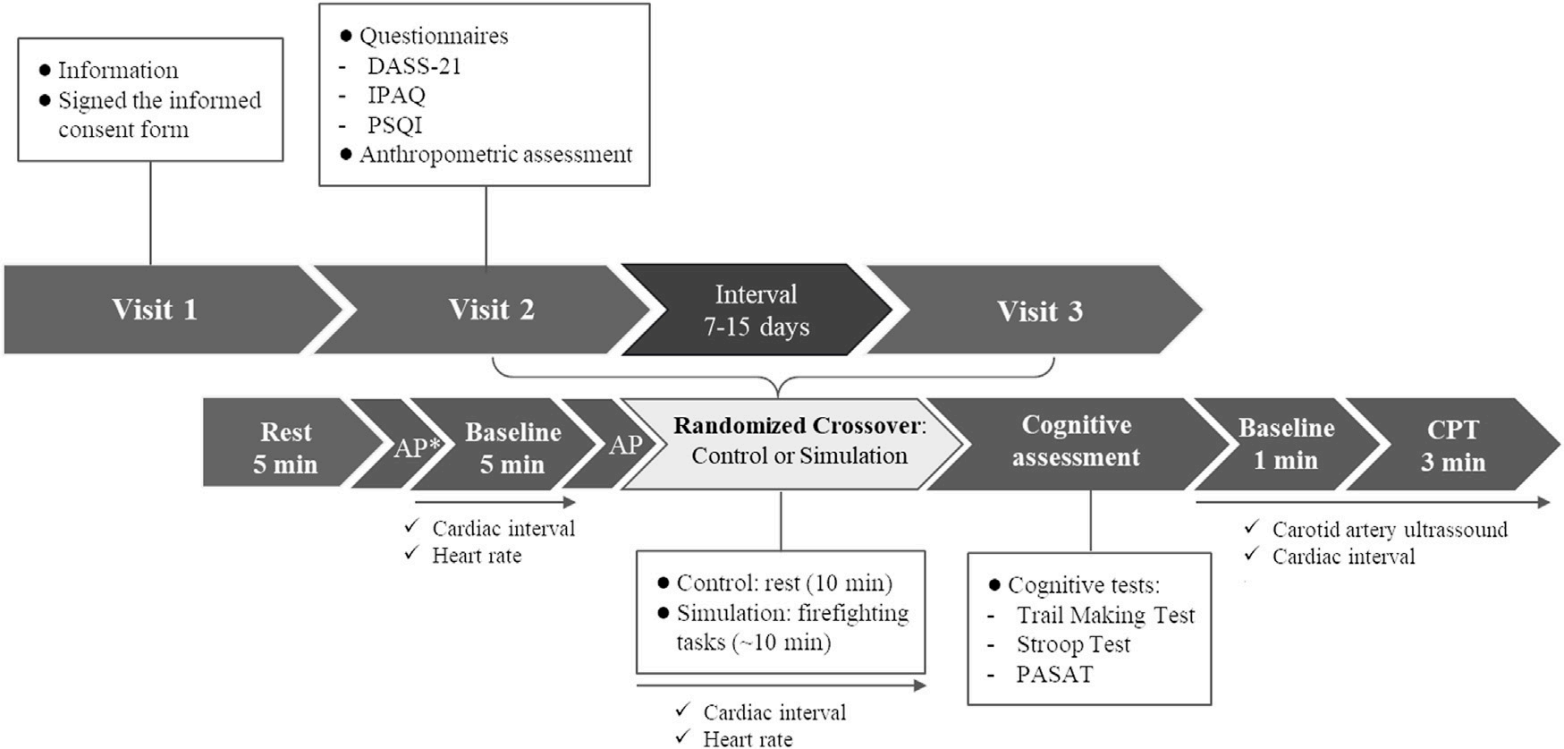
Experimental protocol. AP, arterial pressure; CPT, cold pressor test; DASS-21, Depression, Anxiety, and Stress Scale-21; IPAQ; International Physical Activity Questionnaire; PASAT, Paced Auditory Serial Addition Test; PSQI, Pittsburgh Sleep Quality Index. * AP measured only in the Control Visit for sample characterization.

This experimental randomized crossover study was conducted at the Military Firefighters Corps in Florianopolis, Santa Catarina, Brazil. Participants made three visits to the laboratory. At visit one, participants received information about the research and signed the informed consent form. In visits two and three, the following interventions were performed randomly: a) Firefighting Simulation; b) Control: at rest for 10 min (average Simulation time). Randomization was performed using software with random allocation sequence generation function (Microsoft Excel^®^, Microsoft^®^, Redmond, WA, USA) so that half of the participants started with the Control and half with the Simulation on each data collection day. All the participants performed the interventions in the afternoon starting at the same time. The interval between visits was 7–15 days. In addition, at visit two, before randomization, participants answered questionnaires on mental health, sleep quality, and physical activity levels and underwent an anthropometric assessment (waist circumference, height, and body mass). At visits two and three, before starting the Simulation or Control, the participants remained seated, at rest, for 5 min. Then, they received a call to start the intervention. After the interventions, we assessed cognitive performance using the Trail Making Test, the Stroop Test, and the Paced Auditory Serial Addition Test (PASAT). Finally, the vascular function was assessed through carotid artery reactivity (CAR) to CPT using a Doppler ultrasound. At Simulation and Control visits, the HR and cardiac intervals were recorded continuously using a chest strap connected to a smartphone application for 5 min before and during interventions, and during CPT. Also, the AP was measured through an automated oscillometric device before starting the interventions to evaluate the anticipatory effect. An extra AP measurement was performed only in the Control visit after the rest period to measure the basal AP value for sample characterization (Figure 1). We provided Methods details below.

### 2.3 Firefighting simulation

**FIGURE 2:**
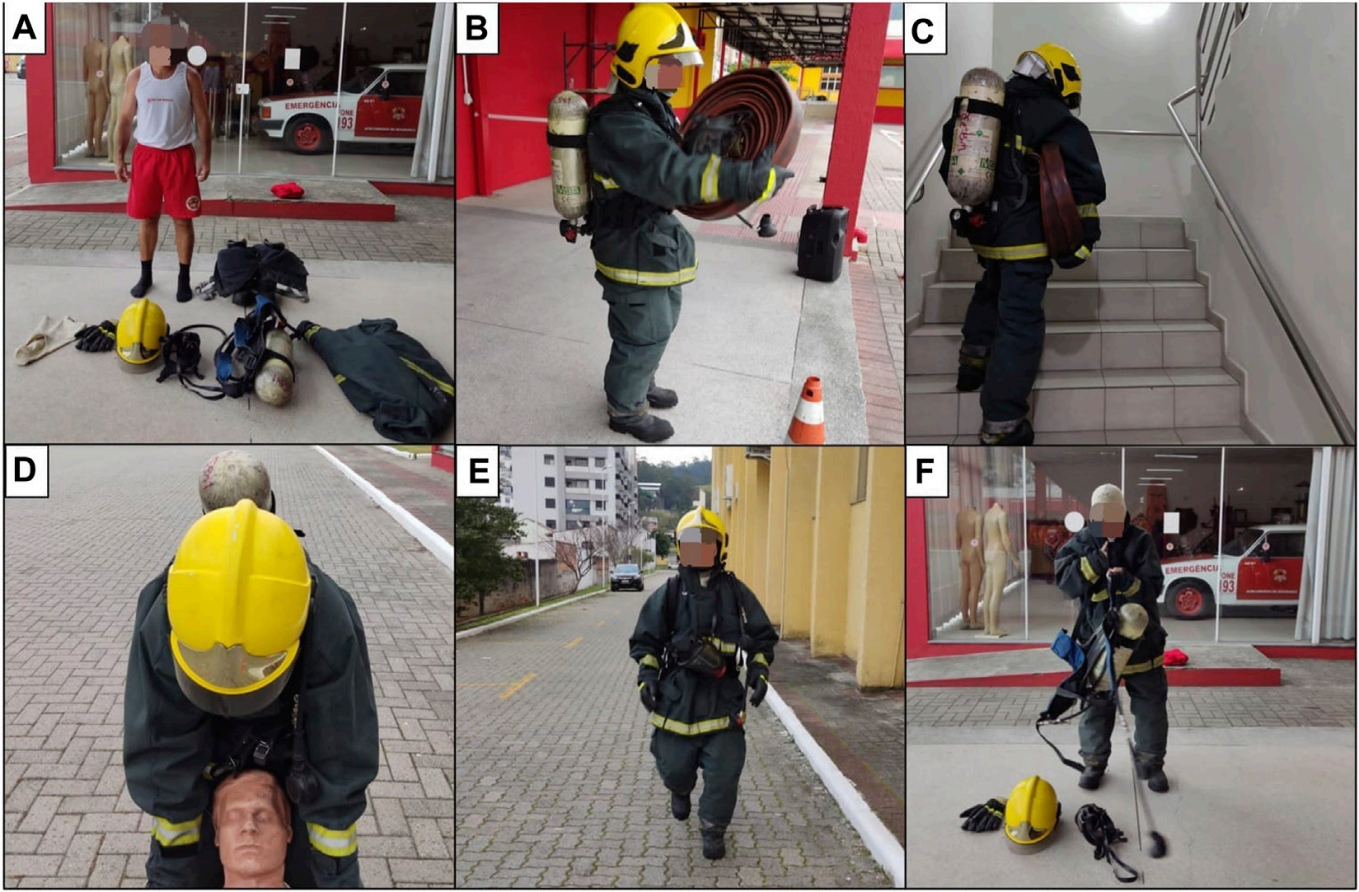
Firefighting Simulation. Getting ready **(A)**; Climbing with equipment **(B,C)**; Rescue **(D)**; Route **(E)**; and Active recovery **(F)**.

The firefighting Simulation was developed based on a training protocol used in the Military Fire Corps of Santa Catarina, Brazil. The Simulation was composed of five firefighting tasks (Figure 2) and was performed in an open environment with a temperature of 21.1°C ± 2.2°C. After collecting the baseline parameters, the participant remained seated until he received a verbal prompt from a designated firefighter to start the Simulation. The tasks were: I) Getting ready. Participants should wear firefighting clothes and shoes and an oxygen cylinder (totaling ∼26 Kg; distance from rest to task I: ∼30 m). II) Climbing with equipment. Fetch a hose roll (∼10 Kg) and go up three flights of stairs taking it. Then, leave the hose and go back to get a second hose, go up the ladder again, leave the hose, and go down the ladder (distance from task I to task II: ∼30 m). III) Rescue. Drag a dummy (∼70 Kg) for 40 m (distance from task II to task III: ∼40 m). IV) Route. Travel 510 m as fast as possible (distance from task III to task IV: ∼40 m). V) Active recovery. Five min consisted of removing firefighting clothing followed by light walking (distance from task IV to task V: ∼40 m).

Participants were instructed to perform the tasks as if they were in a real firefighting situation. Immediately after the Simulation, the Borg Scale (6–20) was used to verify the subjective perception of exertion during the intervention (Borg, 1977; Eston and Williams, 1988).

### 2.4 Questionnaires

To characterize firefighters in terms of sleep quality, physical activity level, and mental health status, we used the Pittsburgh Sleep Quality Index (PSQI), the International Physical Activity Questionnaire (IPAQ), and the Depression, Anxiety, and Stress Scale-21 (DASS-21), respectively.

The PSQI assesses the quality of sleep in the previous month. The higher the score, the worse the sleep quality (Buysse et al., 1998). The final score classifies sleep as good (0–4 points), poor (5–10 points), and presence of disturbance (>10 points). We also used the sleep efficiency provided by the PSQI, which results from dividing the number of hours slept by the number of hours in bed multiplied by 100. The higher the percentage, the greater the sleep efficiency. That is, >85% (score 0), 75%–84% (score 1), 65%–74% (score 2), and <65% (score 3) (Bertolazi et al., 2011).

The IPAQ infers the levels of physical activity from the previous week (Matsudo et al., 2001). Participants who practiced at least 150 min per week of moderate physical activity or 75 min per week of vigorous activity were considered sufficiently active (World Health Organization, 2020).

The DASS-21 assesses the mental health status in the previous week, using the Likert scale, where the number 0 indicates “not applied at all”; 1, “applied to some degree or for a short time”; 2, “applied to a considerable degree or for a good part of the time”; 3, “applied a lot or most of the time”. The scale contains 21 questions, divided into three categories (stress, depression, and anxiety), with seven questions for each (Parkitny and McAuley, 2010; Vignola and Tucci, 2014). The final score is counted and multiplied by two. For depression, it is considered normal (0–9 points), minimal (10–13), moderate (14–20), severe (21–27), and very severe (28+). For anxiety, normal (0–7), minimal (8–9), moderate (10–14), severe (15–19), and very severe (20+). For stress, normal (0–14), minimal (15–18), moderate (19–25), severe (26–33), and very severe (34+).

### 2.5 Cognitive function

The Trail Making Test, Stroop Test, and the PASAT were used to assess cognitive performance.

The Trail Making Test, is an adaptation of Partington’s Test of Distributed Attribution, first developed by John E. Partington (Partington and Leiter, 1949). The test contains two parts, A and B, and evaluates attention, processing speed, cognitive flexibility, and visual search. In part A, the participant has to link a sequence of 25 numbers in ascending order. In part B, the task consisted of interspersing a sequence of letters in alphabetical order and numbers in ascending order, totaling 25 digits (Llinàs-Reglà et al., 2017).

The Victoria version of the Stroop Test, developed by Regard (1981), assesses executive functions through selective attention skills and inhibitory behavior. Three cards were used. With card 1, the participant names the color of colored rectangles. With card 2, he identifies the ink color in which the words are printed. With card 3, the participant names the ink color of words, but the words’ names correspond to different colors, generating an incongruous stimulus (Campanholo et al., 2014). In this test, we used color naming time to assess cognitive performance.

The PASAT, developed by Sampson (1958), consists of a sequence of 61 numbers presented audibly, separated by 3-s of interval. The participant has to add the last number heard to the next and answers the result before the following number is presented (Tombaugh, 2006). The number of correct answers was considered as an assessment criterion.

### 2.6 Cardiovascular parameters

AP was measured in sitting using an automated oscillometric device (Omron HBP 1100, Kyoto, Japan). The acquisition and recording of HR and cardiac intervals were performed using a Polar H10 chest strap (Polar Electro, Kempele, Finland) connected to the Elite HRV smartphone application (Asheville, NC, USA) at 1,000 Hz. The application was previously validated and showed a high correlation with electrocardiogram (Gambassi et al., 2020; Moya-Ramon et al., 2022).

Cardiac interval series were exported from the smartphone application, and HRV analyses were performed using the Kubios HRV Standard software (version 3.5.0; Kubios Oy, Kuopio, Finland). Before the cardiac autonomic modulation analysis, the cardiac interval series were examined, and artifacts and ectopic beats (i.e., non-sinusal beats) were removed using Kubios’s digital filter, considering a maximum acceptable loss of 5% of beats. Since the participants were standing still before the interventions and during CPT, only a very low digital filter was applied. None of the cardiac interval series exceeded 5%.

Time domain indices were analyzed: standard deviation of normal cardiac intervals (SDNN) and the square root of the mean square of the differences between adjacent normal cardiac intervals (RMSSD). In the frequency domain, the time series were transformed into uniformly spaced series by interpolation of cubic splines (4 Hz) and were distributed in overlapping semi-sets of 300 points (Welch periodogram). A Hanning window was used to mitigate the side effects and the interpolated time series had the spectra calculated by the Fast Fourier Transform (FFT) algorithm. The spectra were integrated into the low-frequency bands (LF; 0.04–0.15 Hz), inferring a mixed sympathetic and parasympathetic modulation, but with sympathetic predominance; high frequency (HF; 0.15–0.40 Hz), which correlates with parasympathetic modulation to the heart. Data were presented as natural logarithm (Ln)-transformed and normalized units (n.u.). The LF/HF ratio was analyzed to assess sympathovagal balance (Task Force, 1996; Formolo et al., 2022).

We used a 5-min cardiac interval series to analyze the anticipation of interventions, obtained immediately before starting the Simulation or Control for all time and frequency domain variables. For CPT analyses, we used 1-min cardiac interval series (baseline, 1st, 2nd, and 3rd min of test). In this case, due to the short recording period, we only used the HF (Ln) data, as suggested previously (Task Force, 1996).

### 2.7 Cold pressor test

The CPT was performed with the participants in the supine position on a stretcher, positioned close to one of the right sides, ensuring hand movement without significantly moving the neck. During CPT, the right hand was submerged in cold water (∼4°C). The water temperature was measured with a digital thermometer. Participants were instructed to breathe normally and not speak during the test. After a 1-min baseline, the participants were instructed to place their right hand in the cold water for 3 min (Peace et al., 2020).

### 2.8 Carotid artery reactivity to the cold pressor test

The diameter of the left common carotid artery was assessed by ultrasound (Toshiba Viamo, Toshiba Medical Systems Corporation, Japan) by an experienced evaluator. The image of the common carotid artery, proximal to the carotid bulb, was optimized so that the artery walls were well-defined. Doppler velocity was also recorded at the smallest possible insonation angle (always <60°). Carotid artery diameter was evaluated at rest (1 min) and during CPT (3 min). The mean carotid diameter at rest was considered the basal diameter. During the CPT, the carotid diameter was averaged every 10 s, and the value of the greatest relative change in diameter, above or below the diameter at rest, was used to calculate the CAR in percentage (Peace et al., 2020). The video analyses were performed by a blinded evaluator using FloWave software run in MATLAB 2012b (Coolbaugh et al., 2016).

### 2.9 Statistical analysis

We presented the description of the variables as the mean ± standard deviation of the mean. We analyzed the data using Graphpad Prism 6.0 statistical software (Graphpad Inc.; La Jolla, USA). We verified the data normality using the Shapiro-Wilk test. For Control and Simulation visits comparisons, we used the Student’s T-test with paired measures for normal distribution data and Wilcoxon for non-normal distribution data. Since the Shapiro-Wilk test did not show evidence of non-normality in HF (Ln) data (Control: W = 0.9803, *p* = 0.9661; Simulation: W = 0.9761, *p* = 0.9254), we used the analysis of variance (ANOVA) for repeated measures to assess the HF (Ln) as a function of time during CPT. For all tests, a significance level of *p* < 0.05 was adopted.

## 3 Results

### 3.1 Characterization

Table 1 presents the characteristics of the firefighters, including age, anthropometry, baseline cardiovascular parameters, levels of physical activity, sleep quality, and mental health.

**TABLE 1:** Characteristics of the firefighters.

	Mean	SD
Age (years)	37.8	5.6
Height (m)	1.76	0.08
Body mass (Kg)	82.0	12.6
BMI (kg/m^2^)	26.3	2.5
Waist circumference (cm)	87.6	7.5
WHtR	0.49	0.03
Hemodynamics		
Systolic arterial pressure (mmHg)	127.5	12.0
Diastolic arterial pressure (mmHg)	74.5	10.8
Mean arterial pressure (mmHg)	92.1	9.8
Heart rate (bpm)	67.2	8.1
Physical activity levels		
MVPA (min/week)	138.1	152.1
Sleep Quality		
PSQI Score	7.0	2.2
Sleep efficiency (%)	91.0	5.3
Mental Health		
Depression	4.63	5.15
Anxiety	9.50	7.39
Stress	4.63	3.98

Values are presented as mean and standard deviation (SD); *n* = 16. BMI, body mass index; MVPA, moderate-vigorous physical activity; PSQI, pittsburgh sleep quality index; WHtR, waist-to-height ratio.

### 3.2 Firefighting simulation

Table 2 presents the firefighting Simulation characterization.

**TABLE 2:** Characterization of the firefighting Simulation.

	Mean	SD
Total time (min)	10.0	1.1
Perception of exertion	16.6	1.5
Mean heart rate (bpm)	164.1	10.1
Maximum heart rate (bpm)	183.6	8.7

Values are presented as mean and standard deviation (SD); *n* = 16.

### 3.3 Cognitive performance

**FIGURE 3:**
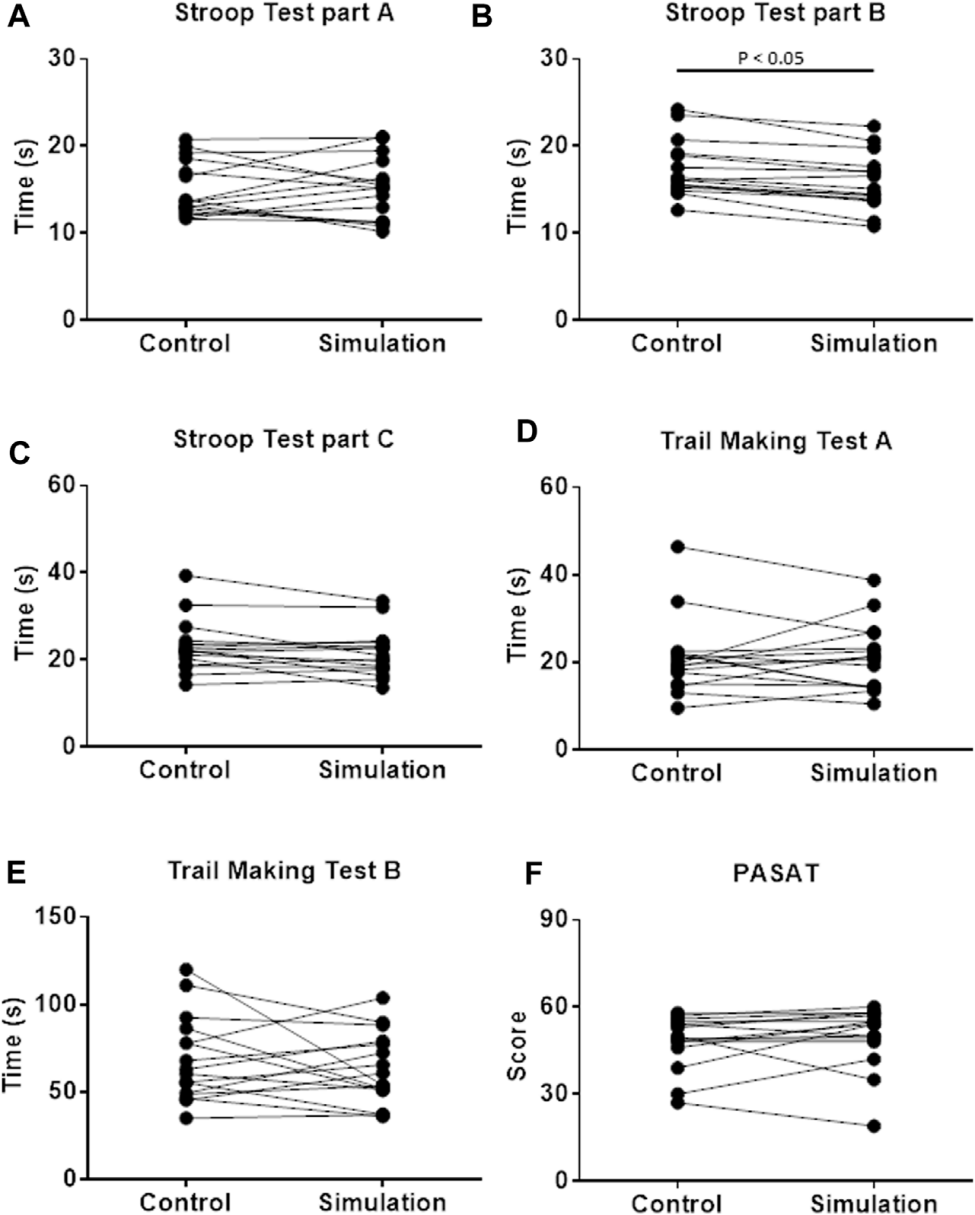
Cognitive performance evaluated by Stroop test **(A–C)**; Trail Making Test **(D,E)**; and Paced auditory serial addition test [PASAT; **(F)**] on Control and Simulation. Wilcoxon test for **(A–F)**; and Student’s t-test for panel E; *p* < 0.05; *n* = 16.

On Simulation, the firefighters performed the Stroop Test, part B, faster than Control. There was no difference between the interventions in the Trail Making Test, PASAT, and Stroop Test, part A (Figure 3).

### 3.4 Carotid reactivity and autonomic modulation response to the cold pressor test after simulation

Analysis of carotid artery reactivity to CPT showed greater carotid dilation in the Control than in the Simulation. In the Control, all participants exhibited carotid dilatation during the CPT. However, in the Simulation, four participants showed vasoconstriction. There was no difference between interventions in the baseline diameter and time-to-peak measurements (Table 3).

**TABLE 3:** Carotid artery reactivity (CAR) to the cold pressor test after Control and Simulation.

	Control	Simulation	*p*-value
Basal diameter (mm)	6.5 ± 0.6	6.4 ± 0.7	0.6427^a^
CAR (%)	6.1 ± 5.7	4.3 ± 8.2	**0.0010** ^b^
Time to peak (s)	105.0 ± 41.4	114.4 ± 32.6	0.5035^a^
Vasodilators (%)	100	75	
Vasoconstrictors (%)	0	25	

^a^ Student’s t-test, values are presented as mean ± standard deviation.

^b^ Wilcoxon test, values are presented as median ± interquartile range; *p* < 0.05; Significant comparisons are highlighted in bold. *n* = 16.

The bold values are the significant comparisons.

**FIGURE 4:**
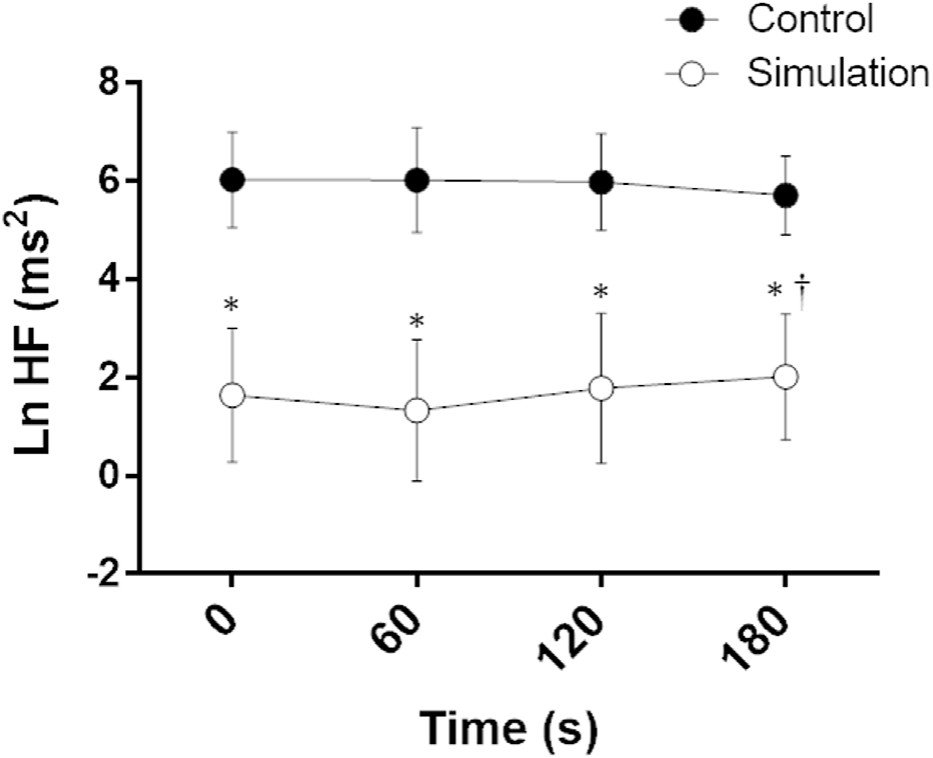
Natural logarithm of high frequency (Ln HF) during the cold pressor test on Control and Simulation. Analysis of variance (ANOVA) for repeated measures; ^*^ Significant vs. Control; ^†^ Significant vs. 60; *p* < 0.05; *n* = 15.

Ln HF analysis showed an intervention effect and an intervention vs time interaction, indicating a lower parasympathetic modulation to the heart during CPT after the Simulation compared to the Control (Figure 4).

### 3.5 Anticipatory effect of simulation on hemodynamics and autonomic modulation

The mean and maximum HR were higher before the Simulation than the Control. Measurements of HRV and AP did not show differences between the interventions (Table 4).

**TABLE 4:** Hemodynamics and autonomic modulation before Control and Simulation.

	Control	Simulation	*p*-value
Hemodynamics			
Mean heart rate (bpm)	69.6 ± 7.2	81.7 ± 6.9	**<0.0001** ^a^
Maximum HR (bpm)	82.6 ± 8.2	98.1 ± 8.9	**<0.0001** ^a^
Systolic arterial pressure (mmHg)	127 ± 14	124 ± 15	0.2958^b^
Diastolic arterial pressure (mmHg)	73.1 ± 10.9	73.1 ± 5.4	>0.9999^a^
Mean arterial pressure (mmHg)	90.2 ± 8.6	91.9 ± 6.1	0.3795^a^
Autonomic modulation			
SDNN (ms)	42.6 ± 12.0	43.6 ± 15.1	0.7791^a^
RMSSD (ms)	33.0 ± 12.1	26.6 ± 16.2	0.1928^b^
Ln LF (ms^2^)	6.9 ± 0.7	7.0 ± 0.8	0.5595^a^
Ln HF (ms^2^)	5.7 ± 0.8	5.5 ± 1.0	0.4450^a^
LF (n.u.)	73.5 ± 12.2	79.8 ± 8.8	0.1017^a^
HF (n.u.)	26.4 ± 12.2	20.1 ± 8.8	0.1019^a^
LF/HF	3.0 ± 4.0	4.1 ± 3.3	0.2312^b^

^a^ Student’s t-test, values are presented as mean ± standard deviation.

^b^ Wilcoxon test, values are presented as median ± interquartile range; *p* < 0.05; Significant comparisons are highlighted in bold. *n* = 16. HF, high frequency; LF, low frequency; Ln, natural logarithm; RMSSD, root mean square of the sum of squares of differences between successive R-R intervals; SDNN, standard deviation of the normal-to-normal RR intervals.

The bold values are the significant comparisons.

## 4 Discussion

In this study, we aimed to assess the effects of a firefighting Simulation on cognitive performance and vascular and autonomic functions in military firefighters. The study’s main findings were: I) cognitive performance was maintained after the Simulation, II) the firefighters had less carotid vasodilation after the Simulation, III) there was lower parasympathetic modulation to the heart during CPT after the Simulation, and IV) although the HR increased, no autonomic anticipatory response was observed before the Simulation.

The firefighters evaluated in our study showed similar cognitive performance in both interventions, performing better only in the Stroop Test, part B, after the Simulation. Previous studies have shown contrasting results. Morley et al. (2012) did not observe changes in cognitive performance in 19 individuals immediately after ∼50 min of exercise on a treadmill with thermal protection clothing in a heated environment. The authors also showed increases in body temperature similar to that observed after fire extinguishment. Thus, it is impossible to state whether it is the ambient temperature or the body temperature that interfered with cognitive performance. However, in the study by Greenlee et al. (2014), 20 firefighters decreased the reaction time decreased (i.e., they were faster) after a Simulation, while accuracy was unchanged in a task that involved information processing, sustained attention, and working memory. The Simulation (∼18 min) included climbing stairs, forced entry, searching a room, and advancing with a hose in a room building containing a controlled fire. Hemmatjo et al. (2020) observed decreases in the cognitive performance on the PASAT in 18 firefighters who underwent simulated live-fire activities for 30 min. The Simulation included passing through live fire, extinguishing the fire using water, and shutting off the fire with a fire extinguisher.

Since the Simulation used in the present study has components of a high-intensity physical exercise, our intervention’s effects may be similar to those of such a stimulus. Thus, we hypothesize that irisin and brain-derived neurotrophic factor (BDNF) may have increased in response to the Simulation, as observed in response to high-intensity exercise (Nygaard et al., 2015; Figueiredo et al., 2019), contributing to the maintenance of cognitive performance. Specifically, it has been demonstrated that irisin acts in the brain, increasing BDNF expression, which increases dopamine content and motivation related to the reward system (Wrann et al., 2013; Zsuga et al., 2016; Ruan et al., 2019). Future studies should investigate the effects of a firefighting Simulation on the irisin and BDNF plasma levels to confirm this hypothesis.

Regarding carotid reactivity to CPT after the Simulation, we observed attenuation of dilation compared to the Control. Again, making a parallel between the firefighting Simulation used in the present study and a high-intensity physical exercise, a previous study corroborates our findings since it demonstrated attenuation of CPT-induced carotid vasodilation after a resistance training session in healthy participants (Heffernan et al., 2017). Immediately before and during CPT after the Simulation, we found less parasympathetic modulation, indicating sympathetic predominance to the heart and, possibly, for the carotid and other pathways. Thus, we hypothesized an increase in noradrenaline, both released by sympathetic postganglionic neurons and circulating (Strobel et al., 1999; Zheng et al., 2014), generated by physical exertion during the Simulation, led to the change in carotid reactivity. Indeed, the stimulation of α_1_ and α_2_ adrenergic receptors in vascular smooth muscle cells by noradrenaline can limit vasodilation and favor vasoconstriction (Pouwels et al., 2019). Knowing that carotid artery dilation during CPT occurs in healthy individuals and the amplitude of dilation is related to cardiovascular risk, Van Mil et al. (2018) sought to understand this phenomenon and mechanism using prazosin, an α_1_ receptor antagonist. The authors found that blocking these receptors attenuated the dilator responses in the carotid and coronary arteries during CPT. Therefore, the α_2_ receptor activation may be related to the Simulation-induced dilatation attenuation. The reduction in endothelial function (Choi et al., 2016) and increase in arterial stiffness (DeVan et al., 2005; Lefferts et al., 2014) induced by acute physical exertion may also help to explain the attenuation of the reactivity of the carotid artery observed in the present study. Further studies are needed to confirm these hypotheses.

In our study, we found an anticipatory effect of HR but not of AP and HRV variables, evaluated in the time and frequency domains. In agreement with our findings, Prell et al. (2020) found no anticipatory effect in a group of firefighters who performed the fire extinguishing Simulation. Bearing in mind that, in some situations, responses to stress seem to decrease with the frequency of exposures (Kothgassner et al., 2021), it is possible that the habituation to the stress of the profession of firefighters has minimized the anticipatory autonomic responses.

Although, in the present study, the firefighters maintained the cognitive performance, the lower carotid vasodilation and parasympathetic modulation to the heart during CPT after Simulation corroborates with the idea of high cardiovascular risk on duty in firefighters. Indeed, previously published data pointing to a high incidence of death from cardiovascular events in firefighters before, during, or after emergency duties (Kales et al., 2007). The firefighters assessed in the present study presented, on average, overweight, symptoms of anxiety and sleep disturbance, in addition to being insufficiently active according to the WHO (Table 1), which recommends at least 150 min/week of moderate-vigorous physical activity (World Health Organization, 2020). Such factors are associated with cardiovascular disorders and may increase the risk of cardiovascular events in physical and mental stress situations, such as firefighting. Indeed, psychiatric disorders and overweight alter the autonomic modulation to the heart (Alvares et al., 2016; Costa et al., 2019), sleep deprivation is associated with cardiovascular diseases (Hu et al., 2021), and endothelial function appears to be impaired in sedentary individuals after acute exercise (Varady et al., 2010).

Our study had limitations. We did not control room temperature during the Simulation due to its execution in an open environment. However, the temperature variation between visits was similar (first visit: 22.56 ± 1.6 x second visit: 21.4°C ± 2.2°C). We also did not control sleep and mood before the interventions. Due to the firefighters’ schedule, our recruitment and randomization were performed for convenience. For instance, although we expected the inclusion of females in the present study, a small number were participating in the sergeants’ course, and none wanted to participate in the research. In addition, our sample (*n* = 16) was restricted to available firefighters in the sergeants’ course. However, previous studies suggest that this number of participants is sufficient to detect differences in cardiovascular function and cognitive performance (Tomporowski et al., 2005; Tsukamoto et al., 2017; Adlan et al., 2018; Pilz-Burstein et al., 2023), our main outcomes. We used a validated assessment for assessing HRV with high similarity compared to an electrocardiogram; however, it is not the gold standard method. Our strengths were the use of vascular function assessment methodology using ultrasound and applied by an experienced professional, in addition to the use of neuropsychological tests applied by a psychologist.

Although simulations are fundamental in research, they limit the extrapolation to real situations. For instance, we did not use fire, which would have generated a warmer environment. Nevertheless, besides the exposure to environmental conditions and pollutants, the cardiovascular risk increases during firefighting due to the activation of the sympathetic nervous system and strenuous physical work (aerobic and anaerobic) (Smith et al., 2016). Indeed, engaging in physical training (12.5%) and responding to an alarm (13.4%) were associated with deaths from heart disease in firefighters in the United States between 1994 and 2004 (Kales et al., 2007). Therefore, in this study, despite the inherent limitations of the Simulation protocol, we included essential components related to firefighters on their duties, including the physical efforts and stress of a call to an occurrence (Smith et al., 2016).

The firefighter profession comprises high cognitive, cardiovascular, and autonomic demands. In this study, we found that a firefighting Simulation did not impair cognitive performance in firefighters. However, the lower carotid dilation associated with changes in autonomic modulation after the Simulation may contribute to greater vulnerability to cardiovascular events in firefighters during work activity. More research is needed to understand the emergence of cardiovascular diseases among firefighters and support public policies for healthcare, wellbeing, and quality of work for this population.

## Data Availability

The raw data supporting the conclusion of this article will be made available by the authors, without undue reservation.
